# GPs’ use and understanding of the benefits and harms of treatments for long-term conditions: a qualitative interview study

**DOI:** 10.3399/BJGP.2020.1027

**Published:** 2021-07-13

**Authors:** Julian Treadwell, Joanna Crocker, Alexander Rushforth, Kamal Mahtani, Trish Greenhalgh

**Affiliations:** Nuffield Department of Primary Care Health Sciences, Oxford.; Nuffield Department of Primary Care Health Sciences, Oxford.; Nuffield Department of Primary Care Health Sciences, Oxford.; Nuffield Department of Primary Care Health Sciences, Oxford.; Nuffield Department of Primary Care Health Sciences, Oxford.

**Keywords:** long-term conditions, multimorbidity, polypharmacy, prescribing, qualitative research

## Abstract

**Background:**

To support shared decision making and improve the management of polypharmacy, it is recommended that GPs take into account quantitative information on the benefits and harms of treatments (QIRx). Quantitative evidence shows GPs’ knowledge of this is low.

**Aim:**

To explore GPs’ attitudes to and understanding of QIRx for long-term conditions.

**Design and setting:**

Qualitative interview study in UK general practice.

**Method:**

Semi-structured interviews were carried out with 15 GPs. Audiorecordings were transcribed verbatim and a framework approach was used for analysis.

**Results:**

Participants described knowing or using QIRx for only a few treatments. There was awareness of this knowledge deficit coupled with low confidence in statistical terminology. Some GPs perceived an absence of this information as an important barrier to optimal care, while others were content to follow guidelines. In the absence of this knowledge, other strategies were described to individualise treatment decisions. The idea of increasing the use of QIRx appealed to most participants, with imagined benefits for patients and themselves. However, potential barriers were described: a need for accessible information that can be understood and integrated into real-world practice, system factors, and communication challenges.

**Conclusion:**

GPs were aware of their knowledge deficit with regard to an understanding of QIRx. Most participants were positive about the idea of increasing their use of QIRx in practice but described important challenges, which need to be considered when designing solutions.

## INTRODUCTION

GPs regularly prescribe treatments for long-term conditions, aiming to improve outcomes for their patients. This might be for a single condition such as isolated hypertension or for multiple long-term conditions such as a combination of hypertension, heart failure, coronary disease, diabetes, and depression.^1^

While GPs understand why they are prescribing (for example, ‘the blood pressure drugs reduce the chance of a stroke’), their understanding of exactly how likely a patient is to benefit from a treatment or experience harm is variable and often inaccurate.^2^^–^^3^

This quantification of the chance of benefit or harms (derived from clinical evidence) can be expressed in a number of ways: absolute risk reduction (ARR), number needed to treat (NNT), relative risk reduction (RRR), or natural frequencies (plain language).^4^ For example, for someone with a baseline 10-year cardiovascular risk of 20%, taking a statin reduces future cardiovascular events by: 7% ARR, NNT (10y) 14, RRR 35%. This means that for every 100 people who take a statin, 13 will have a cardiovascular event over 10 years compared with 20 people out of 100 who do not take a statin.^5^

Knowledge of this kind of information, which is abbreviated in this article to QIRx (quantitative information on the benefits and harms of treatments) enables clinicians to answer questions such as: ‘If I take more tablets for my diabetes, how much does that protect me from diabetes complications?’, or ‘If I take this anticoagulant drug, what is the chance of it causing dangerous bleeding?’.

Thinking about treatment options in this way is increasingly encouraged. For example, the UK Choosing Wisely campaign encourages patients to ask four questions: ‘what are the benefits?’, ‘what are the risks?’, ‘what are the alternatives?’, and ‘what if I do nothing?’.^6^

Maximising treatment benefits and avoiding harm is particularly challenging in the context of multimorbidity and polypharmacy.^7^^–^^8^ The National Institute for Health and Care Excellence (NICE) guideline on multimorbidity^9^ recommends that clinicians should *‘Review medicines and other treatments taking into account evidence of likely benefits and harms for the individual patient and outcomes important to the person’*. This requires an understanding of QIRx for the treatments being considered.

However, international quantitative research shows that doctors’ understanding of QIRx is poor.^2^ The authors conducted a large online survey of UK GPs, which asked them to estimate the ARR for a variety of treatments for common long-term conditions. Only 23% of responses were correct (allowing for ±3% in estimates of ARR), and 65% of GPs reported low or very low confidence in their knowledge.^3^

It is difficult for GPs to find information on QIRx. Clinical guidelines do not offer it,^10^ meaning that clinicians need to seek it from the literature, which many have neither the time^11^ nor expertise^12^ to do. Some online resources do exist, but at disparate locations on the internet, and are not comprehensive in content.^13^^–^^15^

**Table:** How this fits in

Research has shown that doctors, including GPs, often have poor knowledge of quantitative benefits and harms of treatments, such as absolute risk reduction and numbers needed to treat. Yet this kind of information is considered key to shared decision making and optimal management of polypharmacy. This qualitative study explored the attitudes and understanding of GPs in the UK with regard to this issue, and reveals a complex set of behaviours and feelings. These findings will be of interest to doctors wishing to reflect on their own practice, and to authors of guidelines and information resources.

Therefore, if GPs are to use QIRx as part of shared decision making with patients, they need to be supported to acquire this information and integrate it into their practice. This study was part of a larger project to develop an online resource to deliver this kind of information to GPs. To inform this, the authors wished to understand how GPs currently practise, reason, and feel with regard to QIRx. These qualitative aspects remain under-researched despite the extensive quantitative research demonstrating a knowledge deficit. The overarching research question was: what are GPs’ attitudes to and understanding of the quantitative benefits and harms of treatments for long-term conditions?

## METHOD

This study was grounded within a pragmatic research paradigm, suited to the application of the findings to develop practical solutions for use in clinical practice. A patient involvement group was consulted before the research regarding their views on its value and content (see Supplementary Appendix S1). A GP researcher conducted face-to-face semi-structured interviews with 15 GPs in the UK between May and August 2019.

Interviewees were recruited from a pool of 213 GPs who had completed the survey described in the Introduction,^3^ having originally been recruited via widely distributed email invitation. Survey responders had demographic characteristics broadly representative of the UK GP population (see Supplementary Table S1 for details). The inclusion criterion was to be a GP currently practising in the NHS.

From a group of 28 volunteers, purposive sampling of 15 interviewees was used to achieve maximum variation with regard to age, sex, geographical region, rural/urban setting, GP role (principal, salaried, or locum), and deprivation index. It was anticipated that this sample size would generate adequate data, with contingency for further recruitment if this was not the case.

GPs were interviewed in their practices after providing written consent to participate. An interview topic guide (see Supplementary Box S1 for details) was employed and fictional case vignettes (see Supplementary Box S2 for details) were used to prompt discussion when necessary. Interviews were audiorecorded and handwritten field notes were kept. Interviews lasted 1–2 hours.

Audiorecordings were professionally transcribed and pseudonymised. Transcripts were imported into NVivo (version 12) for analysis. All data were stored securely in digital format.

The framework method,^16^ a form of thematic analysis, was used. The framework method was chosen because it allows for both an inductive and deductive approach to analysis, suitable for this study. It provides a structure to consider data within and across interviews, and provides an audit trail linking data, codes, and themes. An initial coding framework was developed by the lead researcher and adjusted iteratively as interview data were indexed. Joint coding of two interviews was undertaken with two other researchers and adjustments to the coding framework agreed. One researcher coded the remaining interviews, then categorised and summarised the data into matrices, each relating to a theme identified during analysis. Further analysis of the matrices by the same researcher identified key elements of data, some requiring linking or re-categorising across themes to develop subthemes and a final narrative.

A process of member checking was undertaken by inviting participants to comment on a near-final version of the article.

## RESULTS

In total, 15 GPs were interviewed. One interview was excluded because the participant had not declared roles conferring significant subject expertise. A diverse sample of GPs was achieved (Table 1). Data saturation was judged to have been reached without further recruitment.

**Table 1.** Participant characteristics

**Characteristic**	***n***
**Sex**	
Female	8
Male	6

**Age, years**	
<30	2
30–39	3
40–49	5
50–59	4

**GP role**	
GP principal	5
Salaried GP	5
Locum GP	4

**Place of original medical degree**	
UK	12
Non-UK	2

**Geographical region**	
North-East England	1
Yorkshire and Humber	1
East of England	1
Greater London	3
South-East England	1
South-West England	4
North Wales	1
East of Scotland	2

**GP description of practice**	
Urban	6
Rural	2
Mixed urban–rural	4
n/a (locum)	2

**Decile of IMD (by practice postcode)^a^**	
1	1
2	2
3	3
4	2
5	0
6	1
8	1
9	1
10	0
n/a (locum)^b^	3

a *Index of Multiple Deprivation (1 = most deprived, 10 = least deprived).*

b *One locum worked regularly in a single practice and was able to provide a postcode. IMD = Index of Multiple Deprivation.*

The findings were summarised in five themes and related subthemes (Box 1) (see Supplementary Box S3 for details of more supporting quotes).

**Box 1.** Identified themes and subthemes

**Theme**	**Subthemes**
Descriptions of GPs’ current use of QIRx	Examples of successful use
	‘Partial’ use of QIRx: risk scores as thresholds but without knowledge of subsequent risk reduction
	Internalised non-numerical ideas of the value of treatments

Discussion of the lack of use of QIRx	Awareness of a knowledge deficit
	An absence of accessible information in a context of information overload
	Low confidence in statistical terminology
	Competing drivers to clinical practice

Making decisions in the absence of QIRx	Working with non-numerical, internalised ‘knowledge fragments’
	Using knowledge of physiology and extremes of age or risk
	Using non-numerical heuristics
	Taking into account patients’ characteristics
	Employing qualitative communication styles to convey non-numerical estimates of risk

GPs’ attitudes and feelings about the use or non-use of QIRx	Positive expressions of the value of QIRx in current practice
	Relative contentment with not using QIRx for some
	Negative impact on patient care due to a lack of understanding of QIRx
	Negative emotions arising from challenges in this area

GPs’ views on possibly increasing the use of QIRx in the future	Imagined benefits of increasing the use of QIRx
	Anticipated barriers to increasing the use of QIRx

### GPs’ current use of quantitative information on the benefits and harms of treatments

Only a small number of examples were given of the use of QIRx, typically only one or two per GP.

The prescribing of statins for the prevention of cardiovascular disease was the only area where any participants confidently described a numerical risk reduction (treatment benefit) for a patient. This information was acquired from decision support tools either on paper, downloaded, or built into clinical systems *.*

Quantitative information about specific treatment harms was described by a few, for example, major risks associated with hormone replacement therapy (HRT), or bleeding risks associated with anticoagulants. This information was usually derived from an external resource such as a patient information leaflet. GPs seemed to value this information and confidently apply it in practice *.*

Numerical risk scores calculated to estimate patients’ risk of future health problems, such as QRISK^17^ or FRAX,^18^ were discussed by many. These could be regarded as an aspect of QIRx, and were often discussed without a subsequent understanding of how treatment might reduce this risk, that is, the actual benefit of the treatment. Instead, they functioned as a simple treatment threshold:

*‘NICE … talk about if your risk is over 10% you are likely to benefit … So, I tend not to take an ownership of the recommendation. I say “this is what’s recommended”*

(Interviewee 4)

One GP described using a risk threshold to support a decision they had already made on the (non-numerical) basis of various risk factors.

Some described using internalised ideas about the value of treatments that were non-numerical or imprecise:

*‘So, I think, “well, can I take him off his simvastatin?” … I would think number needed to treat, 500 or something, whatever it is … On the other hand, there’s atrial fibrillation, risk of stroke, and that’s extremely high … so, I would be a lot more trigger happy to start on a NOAC* [novel oral anticoagulant] *or a warfarin, and that’s got a very low NNT, which I can’t remember.’*

(Interviewee 2)

### The lack of use of quantitative information on the benefits and harms of treatments

GPs described their awareness of a lack of this specific kind of knowledge, one framing it as ‘missing’ information:

*‘I know that I’m a little bit in the … or perhaps massively in the dark about this.’*

(Interviewee 1)

*‘But actually … I think it is missing … if you’re making a decision about starting someone on a tablet for potentially the rest of their life … it’s important you get that decision right.’*

(Interviewee 6)

Descriptions were given of a lack of QIRx in the context of particular conditions and treatments, for example hypertension, type 2 diabetes, and fracture prevention.

A number of barriers to the use of QIRx were described, including, importantly, a lack of easily available information. One GP described an online tool on the benefits of statins and speculated about the possibility of a similar tool for hypertension treatment:

*‘I haven’t used these* [decision aids] *for hypertension though … I’m not sure they’re there; they might be … but they might not be as clear cut*. *‘*

(Interviewee 1)

On the other hand, the challenge of retaining such information in a context of information overload was described.

Many GPs reported low confidence in statistical terminology (such as ARR and NNT), sometimes implying that they imagined this to be too difficult, or too specialist to be part of core GP skills:

*‘It’s funny because stats were my thing before … it wasn’t an area that frightened me, like I like numbers … so it’s a bit shocking when you think about it … but I can’t even remember what the terms mean.’*

(Interviewee 3)

Wider drivers to clinical practice were described that shape decisions in the absence of QIRx, or might act as barriers to its use were QIRx available. These included clinical guidelines, performance measures such as the Quality and Outcomes Framework (QOF), a desire to conform to normative practice, and fear of adverse outcomes, including medico-legal fear:

*‘I’m looking at their numbers before they come in and thinking, “Oh, right, we need to get that one better.” Part of the decision making is driven by QOF and the numbers that they are set in in my computer.’*

(Interviewee 1)

*‘I … have been beautifully indoctrinated for 20 years that “thou must have a lower blood pressure”* . *’*

(Interviewee 14)

*‘I suppose in the back of my mind someone somewhere has said “this is a good medication”. I don’t want to be the one to go against that; him have another heart attack and someone say, “Well, why did the GP stop that?”.’*

(Interviewee 12)

### Making decisions in the absence of quantitative information on the benefits and harms of treatments

GPs are still required to make decisions with patients about treatments even in the absence of knowledge of QIRx. A variety of strategies were reported drawing on non-numerical, internalised ‘knowledge fragments’ about the value of treatments:

*‘So, after a STEMI* [ST-elevation myocardial infarction] *, I think bisoprolol is the most evidence-based … I can’t remember the numbers but … exam question … bisoprolol was the one on the multiple choice that you tick is the most useful* . *’*

(Interviewee 12)

*‘So I tell my patients … their most important drug is metformin, out of your diabetes drugs … that’s not only helping your sugars but it’s also helping your heart and your vessels.’*

(Interviewee 3)

Some GPs drew on their knowledge of physiological mechanisms to support decisions, for example, managing the urateraising effect of thiazides in a patient with gout and hypertension. Another considered the relative short-term risks of stopping anticoagulant or blood pressure-lowering drugs:

*‘So, if I stop the* [anticoagulant] *drug and* [the blood] *becomes thicker … maybe it won’t get through. That might have a more … immediate effect. But I feel like the blood pressure has more of a longer-term effect.’*

(Interviewee 1)

Framing thinking around extremes of age or risk (and therefore higher and lower chance of benefit or harm) was a mechanism described by many:

*‘So, if you’re looking at a 90 year old who’s on a statin, you know, the chances are they’re not going to live for very many years … the gains are always going to be marginal.’*

(Interviewee 6)

Some described using non-numerical risk comparators as communication tools. Examples were comparing the risk of breast cancer from HRT (small) to the risk associated with moderate alcohol consumption (larger), or the risk of osteonecrosis of the jaw from bisphosphonates with getting hit by a car.

Some described internalised heuristics from previous teaching or experience:

*‘It’ll be more gut instinct which is really non-numerical and not very medical, but it‘s a synthesis of the information and experience you’ve had, I guess, over the years.’*

(Interviewee 14)

The GPs’ understanding of their patient’s characteristics and medical history could be integrated to guide treatment choices. In one example, the GP considered an individual’s tendency to develop side effects. In another, the GP considered the risks associated with gliclazide for a frail patient with diabetes, and decided (without numerical estimates) that these outweigh the benefits, taking into account the possible non-applicability of trial evidence to this individual:

*‘You have to base it on the patient in front of you, not on population studies … she’s approaching her eighties … causing hypos is a bit more risky than running a bit high on the sugar at her age maybe. You know, she’s living on her own and she has a hypo, she falls, breaks her hip … So I’d be very keen to, at some point, tail off the gliclazide.’*

(Interviewee 2)

Sometimes, a lack of quantitative information was hidden within qualitative communication styles, such as here, where the GP discussed the choice to treat mild hypertension:

*‘I’d probably* [say] *“Overall, over the next 10/20 years if we keep this controlled we’re likely to reduce the risk of … ” but I’m not going to give them numbers because I haven’t … got easy numbers to give them. And I would try to convey … “There’s definitely a benefit, but if you don’t want to we can monitor things …” And it’s all … fluffy and communicative … very much nonscientific and non-numerical … because I haven’t got the numbers on the tip of my tongue.’*

(Interviewee 14)

### GPs’ attitudes and feelings about the use or non-use of quantitative information on the benefits and harms of treatments

Some GPs discussed positive aspects of their current use of QIRx, such as supporting informed choice or medico-legal confidence.

Regarding the status quo of relatively little use of QIRx, some GPs expressed a reasonable degree of comfort, being happy to trust and follow guidelines or accepted practice without taking further ownership of decision making:

*‘Even if you told me what the number needed to treat or number needed to harm for any given drug is, I’d forget it. So I just need to rely on guidelines and formularies … to help guide me.’*

(Interviewee 9)

Others felt less comfortable, expressing concerns about over-treatment or a lack of personalised care:

*‘My feeling is that we treat a lot of people according to thought-free algorithms because they’ve got a condition that feeds in the top end … without really having a sense of how important what we’re doing is.’*

(Interviewee 14)

*‘It’s very apparent that for people … who are getting older and frailer and have comorbidities, suddenly you’re way over-treating or massively increasing the complexity of their life.’*

(Interviewee 14)

Negative emotions clearly arose for GPs when thinking about this aspect of their practice, including fear of adverse health outcomes and a degree of shame about their perceived knowledge levels:

*‘I think there’s a big thing amongst GPs about how they’re “just GPs” and there’s a kind of collective hidden shame in not knowing about this stuff … and just don’t assume anything about the level of competence of GPs because we’ve forgotten everything. I’m terrified about how little I know having* [been interviewed] *today.’*

(Interviewee 13)

### GPs’ views on possibly increasing the use of quantitative information on the benefits and harms of treatments in the future

Considering whether a hypothetical new information resource delivering information on QIRx would be helpful, including whether they would actually like to increase their use of QIRx at all, most GPs expressed positive views, imagining benefits for their patients and themselves:

*‘The information’s helpful for some patients because they want it, but it would also be helpful for me, so I feel a bit more like I’m on firmer ground about what I’m actually suggesting.’*

(Interviewee 15)

*‘So the patient perspective might be, “Well, that doesn‘t change my risk much”, whereas going from 30% likely to have a stroke to 20% … they might see that as more of a significant finding.’*

(Interviewee 12)

However, concerns were raised about the suitability of sharing QIRx with particular patients, and whether it would actually affect their choices. Other concerns included introducing QIRx into the workflow, and the potential for distraction or its place among conflicting priorities:

*‘The more I do this, I actually think we respond to expectation: number one of patients, number two of hospital consultants, number three of what we think we can manage, number four … government and CCG* [Clinical Commissioning Group] *controls. And the rationality in medicine is probably number five.’*

(Interviewee 5)

## DISCUSSION

### Summary

The GPs interviewed only described using QIRx for a few treatments, such as considering the treatment benefits of statins and for some specific treatment risks. They were aware of their knowledge and confidence deficit in this area, with mixed attitudes regarding this. Some perceived it as an important gap in their ability to provide optimal care whereas others were content to follow guidelines. Often, an individual GP would hold both these perspectives.

Instead the GPs used a variety of strategies to make treatment decisions, drawing on their clinical knowledge and understanding of individual patients.

Regarding the idea of increasing their use of QIRx, most GPs were positive, imagining benefits for patients and themselves. However, barriers to such a change in practice were described. These included pressure to conform to clinical guidelines and performance measures, perceptions of normative practice, and medico-legal fear. GPs need accessible, understandable information on QIRx that can be integrated in their complex, time-poor practice.

### Strengths and limitations

To the authors’ knowledge, this is the first qualitative study to specifically explore GPs’ understanding and use of QIRx.

The sample of participants had a broad range of characteristics reflective of the wider GP population. Some bias may have occurred as a result of participant self-selection, attracting participants with an above average level of interest in the topic. The sample did not appear to be unusually confident in their use of QIRx but all GPs might not share the same degree of positivity regarding an increase in its use in practice.

The setting of interviews in GPs’ surgeries allowed reference to computer systems and information resources, acting as valuable prompts to discussion.

That the interviewer was also a GP supported spontaneous expression and understanding. A limitation might be that given the interviewer’s interest in the subject, participants may have shaped their answers to what they imagined was ‘correct’. The lead researcher was mindful that his previous assumptions might affect the analysis. These potential sources of bias were mitigated by an interview guide employing positive and negative framing of questions, dual coding of early transcripts with non-clinicians, member checking, discussions with academic supervisors, and ongoing reflection.

The framework method involved repeated cycles of analysis and created a robust audit trail linking data to conclusions.

### Comparison with existing literature

Existing explanations for why knowledge deficits on QIRx exist include biased or oversimplified information from researchers, industry, or guideline producers.^2^^–^^3^ Many also arose in this study, including lack of access to information, the dominance of system drivers such as performance measures, and medico-legal concerns.

The qualitative literature on GPs’ management of multimorbidity and polypharmacy describes challenges faced by GPs who feel poorly equipped,^19^ and even helpless^20^ in this area. Difficulties applying single-condition guidelines to individual patients and sharing decisions about treatment in the absence of applicable evidence are common themes, as is medico-legal fear.^21^ One strategy described to deal with this is ‘satisficing’:^22^ combining hunches, best guesses, and negotiating compromise in an attempt to offer optimal personalised care.^23^ These findings are echoed in the present study; however, the role of QIRx is mentioned only rarely and superficially in the referenced literature.^19^^,^^24^^–^^25^

Similarly, literature on GPs’ relationships with guidelines describes their reservations, including doubt about their applicability to individuals; tension between doctor experience, patient preferences, and guideline recommendations; and time and communication constraints.^26^^–^^27^ Specific discussion of QIRx does not feature, although in the last decade expert commentators have highlighted the lack of information on QIRx in guidelines and called for improvements.^10^^,^^28^^–^^31^

Much work has been done in the field of formal shared decision making to introduce QIRx into consultations.^32^^–^^33^ This process typically involves a patient-facing resource containing information on QIRx for a single condition, supported by an implementation plan and consultation model. However, uptake of this strategy has been poor, with multiple barriers described, including institutional and organisational issues, time limitations, and complex interpersonal dynamics between clinicians and patients.^34^

### Implications for practice

The findings of this study will inform a participatory co-design process to develop a novel online resource intended to support GPs in making shared decisions with their patients. The idea that GPs acquire knowledge about QIRx and integrate it into their practice is an attractive one, with obvious benefits. The statements and practice examples in this study indicate that GPs might be enthusiastic about this.

In this study the use of QIRx was described with regard to statin prescribing and in giving information on some specific treatment risks. What unites these examples is that GPs had access to usable information, and the option for patients to take these treatments (statins, bisphosphonates, and HRT) or not has been the subject of mainstream debate. Given the right information and a sense of ‘permission’ to offer choice, the GPs seemed to have found a way to integrate this information into their consultations.

Their practice of shaping decisions based on even imprecise ideas of benefit and harm, drawing on their tacit knowledge and understanding of individual patients, suggests there may be an appetite to enhance this with a sharper understanding of QIRx.

Use of QIRx might resolve some longstanding problems regarding multimorbidity, polypharmacy, and guidelines. Improved ability to balance benefit and risk would support discussions with patients about developing individual and personalised treatment plans. Better understanding of clinical evidence might increase GPs’ trust in guidelines, bridging the gap between practitioner independence, experience, and apparently rigid guideline recommendations. It is remarkable how little this has been explored.

However, it is not an easy challenge: the translation of evidence into practice is notoriously difficult.^35^ The GPs interviewed imagined a number of barriers: time constraints, interruption to consultation flow, clinical complexity, and variable applicability to patients. There will be a need to further develop risk communication and consultation skills to underpin this process.^36^^–^^37^ The quantitative information itself is only one element of many in the choices patients may make in partnership with doctors. Establishing patients’ values and preferences is critical to shared decision making,^38^ and individuals may have priorities that override rational risk-based decision making. Despite wishing to exercise choice and have their opinions valued, many patients still want their doctor to make final treatment decisions.^39^^–^^40^

GPs use online resources regularly,^41^ so this would seem the obvious route to deliver information on QIRx, either integrated into existing clinical guidelines or via a novel resource. Such resources need careful user-centred design, cognisant of the demands and time pressures under which GPs work, their ways of accumulating knowledge (described by Gabbay and le May as ‘mindlines’^42^), and their levels of statistical literacy.^43^^–^^44^

A challenge to overcome is that much of the quantitative evidence on the benefits and harms of treatments for long-term conditions is derived from relatively young, healthy participants in clinical trials,^45^ or populations not representative of primary care practice.^46^ Any information resource would, therefore, need to be open about the origin and applicability of data, communicating this in a way that supports shared understanding of the evidence between doctor and patient,^47^ while avoiding inappropriate simplification and certainty in a context where multimorbidity and polypharmacy are the norm.
